# The Modern Treatment of Heart Disease

**Published:** 1895-06-08

**Authors:** 


### The Hospital

AN INSTITUTIONAL JOURNAL OF

The Medical Sciences and Hospital Administration,

WITH

Vol. XVIII.—No. 454.] AN EXTRA NURSING SUPPLEMENT. [June 8, 1895.

### The Modern Treatment of Heart Disease.

Few things have retarded the progress of medicine more than the concentration of the medical mind upon the drug treatment of disease. Hydropathy, massage, baths, dietetic "cures" of various sorts, electrical treatment, and all kinds of movement cures, although not absolutely banned, have all been looked upon as just tainted with such a degree of suspicion of quackery as to prevent their being cordially received among orthodox measures.

In no department has this feeling had such influence as in cardiac therapeutics. Digitalis, convallaria, and strophanthus have been poured in as tonics to the heart in the hope of regulating its action or increasing its force, while other drugs have been employed as purgatives to lessen the mass of blood to be driven, as diaphoretics to increase the area through which it circulates, or as expectorants in the hope of easing the flow through the lungs, and a multitude of vaso-dilators have been employed with the aim of lessening arterial tension, and thus, even if only temporarily, of relieving the over-labouring heart. Meanwhile, the vast field of therapeutics covered by the various applications of baths, exercise, and friction have, till quite lately, been left to either to people outside the pale, or to free lances who, while accepted at watering-places as, in a sense, proper to such localities, were hardly taken seriously by the rest of the profession. During recent years, however, perhaps coincidently with a greater and more accurate physiological knowledge, these more general measures have come into wider use, and have tended largely to displace the older plan of driving the heart by drugs.

Oertel's method of treating chronic heart disease by means of graduated exercises is now a recognised form of "cure" at many Continental spas. There are many, however, who, while by no means denying the benefits so derived, attribute them to the fact that the patients are in many cases suffering as much from ill-conditioned blood and tissues as from enfeebled heart; and it has seemed to some more reasonable to believe that the exercise relieves the morbid condition of the blood, clears out the uric acid with which the system is imagined to be crammed, and lessens the general "stodginess" of the patient, than that a heart already incapable of doing its full work is cured by making it do more. There are many cures, however, which are not explicable on this simple hypothesis, and as year by year patients who before could hardly puff across a London square are seen going cheerfully through the daily treadmill of a "mountain cure," it becomes obvious that there is still something unexplained.

The effect of bathing has always been more or less of a mystery, and yet we found patients with dilated, flabby hearts and incipient dropsy going to Matlock or to Ilkley, or to the various watering-places abroad, submitting to baths, and rubbings, and mustard packs, and coming home with their lease of life renewed. Now we have the Schott system as carried out at Nauheim, a combined system of exercise and bathing, which appears capable of giving relief to a class which are obviously not of the mere "stodgy" or gouty type, but are in many cases examples of progressive dilatation from organic disease; the beginning of the end. Any method which can bring these cases back to a condition of enjoyable and reasonably useful life is worthy of our serious and respectful consideration. In seeking for the *modus operandi* of this method we must first remember that man, having during the processes of his evolution been a naked or at the best an imperfectly clothed animal, has retained the habit of preventing the loss of body heat by letting as little blood as may be circulate through his skin and subcutaneous tissues; also that while muscles are at rest the circulation is at its minimum. Under these circumstances it is not difficult to see that the average sufferer from chronic heart disease, resting, moping, walking slowly, careful at every turn to avoid either exertion or excitement, carries about with him two enormous vascular areas, the cutaneous and the muscular, either of which is capable of absorbing a large proportion of the blood of the body and of thus reducing the pressure against which the heart expends its energy. Of these things the heart knows nothing. Its daily duty is to keep the aorta full, but the effort it puts forth in doing this depends upon the difficulty or the ease with which the blood flows out at the other end, and thus by enlarging the area into which it flows the work of the heart is lightened.

There is but little doubt that this is the explanation of the good results which, in suitable cases, follow the Oertel treatment and the Schott treatment, and which for years past have now and then followed the use of packs and hydropathy in serious heart disease. Quite a literature is springing up on this subject. Special watering places, special men, special methods, are advocated in one journal or another; but underlying all these recommendations is the broad principle that, without drugs to force the heart, or drugs to purge, or sweat, or act upon the kidneys, the mere bringing into functional activity of large vascular areas lessens the tension against which the heart is working, and, by enabling it to contract more fully, at once diminishes its dilatation. The enthusiasm of the individuals who have devised these methods of treatment has properly and justly drawn to foreign watering-places crowds of anxious patients; but there seems no reason why, at any English sanatorium which possesses pure air, saline baths, and careful doctors, this treatment should not be carried out with complete success. But, independently of this, if it is once recognised that relief to the heart can be obtained by opening out these great vascular areas, and that this can be done by simpler methods than by the exhibition of drugs, great benefit will accrue to one of the most pitiable classes of invalids.

